# Redox Conductivity of Current-Producing Mixed Species Biofilms

**DOI:** 10.1371/journal.pone.0155247

**Published:** 2016-05-09

**Authors:** Cheng Li, Keaton Larson Lesnik, Yanzhen Fan, Hong Liu

**Affiliations:** Biological and Ecological Engineering, Oregon State University, Corvallis, OR 97333, United States of America; US Naval Reseach Laboratory, UNITED STATES

## Abstract

While most biological materials are insulating in nature, efficient extracellular electron transfer is a critical property of biofilms associated with microbial electrochemical systems and several microorganisms are capable of establishing conductive aggregates and biofilms. Though construction of these conductive microbial networks is an intriguing and important phenomenon in both natural and engineered systems, few studies have been published related to conductive biofilms/aggregates and their conduction mechanisms, especially in mixed-species environments. In the present study, current-producing mixed species biofilms exhibited high conductivity across non-conductive gaps. Biofilm growth observed on the inactive electrode contributed to overall power outputs, suggesting that an electrical connection was established throughout the biofilm assembly. Electrochemical gating analysis of the biofilms over a range of potentials (-600–200 mV, vs. Ag/AgCl) resulted in a peak-manner response with maximum conductance of 3437 ± 271 μS at a gate potential of -360 mV. Following removal of the electron donor (acetate), a 96.6% decrease in peak conductivity was observed. Differential responses observed in the absence of an electron donor and over varying potentials suggest a redox driven conductivity mechanism in mixed-species biofilms. These results demonstrated significant differences in biofilm development and conductivity compared to previous studies using pure cultures.

## Introduction

The efficient extracellular transference of electrons is critical to the functioning of many biological processes in both natural and engineered environmental systems [1–3]. Much of our current understanding of extracellular electron transfer in these environments is largely based on the indirect transfer of small molecules such as hydrogen and formate, but recent evidence suggests that extracellular electron transfer through electrical current is prevalent [1,4–6]. In diffusion-limited environments, such as biofilms and sediments, direct extracellular electron transfer via electrical currents could offer significant advantages over small molecule exchange. It is likely that physical connections in the form of aggregates and biofilms are often established in order to support electrical interactions between microorganisms and extracellular electron acceptors including other microorganisms and electrodes [1,4,6–7]. Biofilms and methanogenic aggregates associated with microbial fuel cells (MFCs) and anaerobic digesters have been found to exhibit electrical conductivity further reinforcing the hypothesis that interactions via electrical currents are a critical component of these environments [8–10].

Biofilm and aggregates conductivity is often associated with the presence of specific microbial species that use direct extracellular electron transfer as a primary means of respiration [6,8–11]. This includes both *Geobacter sulfurreducens* and *Shewanella oneidensis*, two species known to produce conductive extracellular appendages known as microbial nanowires. The conductive properties of many of these microbial nanowires have been characterized in detail using techniques such as atomic force microscopy [12–14]. However, biofilms of *G*. *sulfurreducens* are the only pure cultures of which conductivity has been examined to date with conductivity up to 5000 μS cm^-1^ having been previously reported [8].

The conduction mechanism of nanowires and biofilms is currently not well-established and is still being explored. Though the conductivity of *S*. *oneidensis* nanowires appear to be dependent on the presence of redox cofactors like c-type cytochromes that are typically associated with extracellular electron transfer, the nanowires of *G*. *sulfurreducens* appear to have a conductivity that is independent of redox cofactors [8,12,14–15]. Some experimental evidence suggests that the microbial nanowires of *G*. *sulfurreducens* possess delocalized electronic states representing a metallic-like conductivity that is conferred to whole biofilms [16]. However, other studies have refuted this theory and indicated that electron transfer in whole biofilms of *G*. *sulfurreducens* proceeds through a concentration gradient-driven electron transfer process involving localized redox cofactors referred as electron hopping [17–20].

Conductive properties have also been recognized in various mixed consortia including methanotrophic aggregates in which electron transfer is hypothesized to proceed through multi-heme cytochromes [21]. Conductivity is also a recognized property of mixed-species MFC biofilms enabling multilayer cell stacking and efficient cell-electrode contact conducive to high power outputs and coulombic efficiencies [9,22–23]. A conductivity of 250 μS cm^-1^, around 5% of the pure culture biofilms of *G*. *sulfurreducens*, was observed in a *G*. *sulfurreducens*-dominated (52% *Geobacter* spp.) mixed species MFC biofilms. However, additional characterization of mixed-species communities in terms of their extracellular electron transfer mechanisms has yet to be performed. Because several microbial species are capable of producing various conductive proteins/redox cofactors the combination of different species could affect the overall conductive characteristics of mixed species biofilms [12–14,20]. Future enhancement of microbial electrochemical systems (MESs) could depend on the elucidation and optimization of the extracellular electron transfer processes within mixed-species biofilms [5,23–25].

In the present study, a gold-coated split-anode design was used to examine the conductive behavior of high-power, mixed-species MFC biofilms over an extended range of anode potentials (-600–200 mV vs. Ag/AgCl) in both the presence and absence of an electron donor [8]. Results demonstrated significant differences in conductivity compared to previous studies using pure cultures and provides evidence for redox driven conductivity in mixed-species biofilms of MFCs.

## Materials and Methods

### Anode preparation

A split-anode design modified from a previous study was developed for the in situ evaluation of biofilm conductivity [8]. A water resistant adhesive (Loctite, Düsseldorf, Germany) was applied to standard weighing paper (Schleicher & Schuell, Inc., Keene, NH, USA) to provide rigidity. Adhesive laden paper was then cut into a circle with area of 7 cm^2^. An electrically conductive gold film (approx. 5 μm) was then applied to the adhesive layer by an Cressington 108 auto sputter coater (Cressington Scientific, Watford, UK). The gold film layer was then cut down the center by an ESI 5330 UV Laser machine (Electro Scientific Industries, Inc., Portland, OR, USA) to create a non-conductive gap of 50 μm. Resistance measurements confirmed that the two pieces of the anode were electrically separated (R_gap_ > 10^10^ Ω).

### Microbial fuel cell design and operation

**Fig 1:**
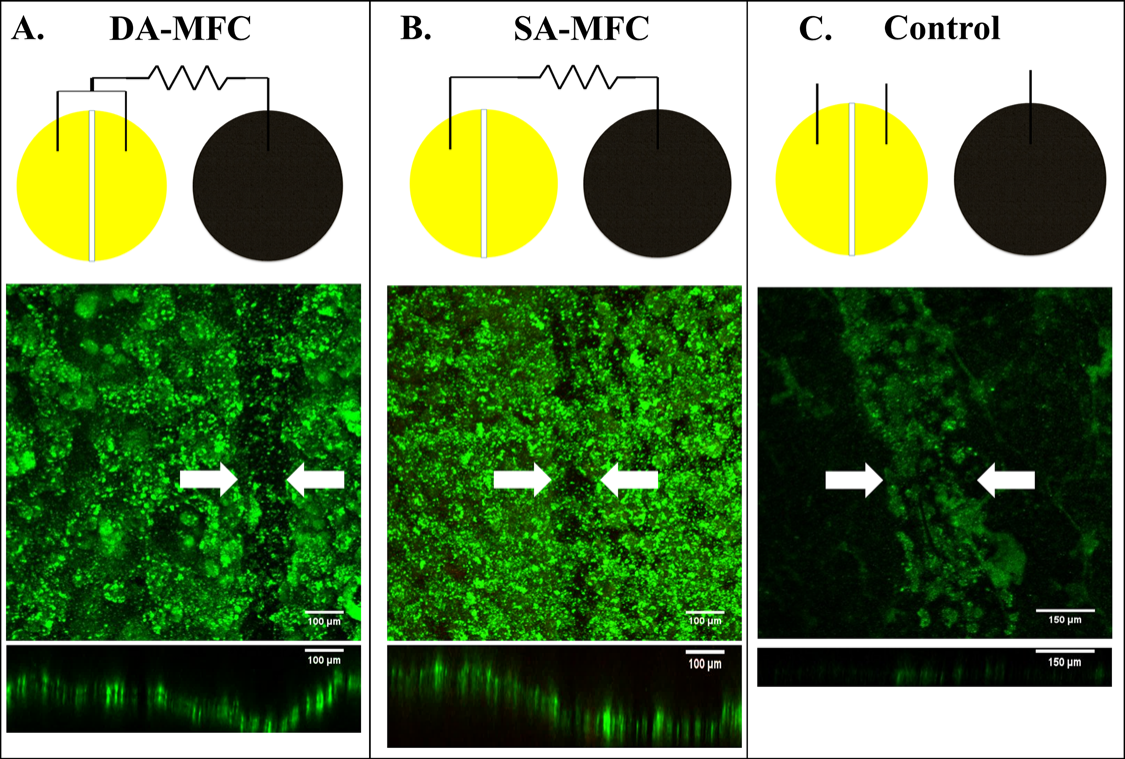
Top down CLSM images and schematic of MFC setup used for culture of exoelectrogenic biofilms. (A) Anode from DA-MFC with 50 μm non-conductive gap, (B) Anode from SA-MFC with 50 μm non-conductive gap, and (C) Electrode from control MFC with 300 μm non-conductive gap. Arrows indicate the location of non-conductive gap. Frame size = 8.1 X 10^−3^ cm^2^.

Single-chamber, air-cathode MFCs with gold-coated split-anodes were used to develop biofilms on the anode surface. Carbon cloth/activated carbon cathodes were fabricated following previously developed protocols [26]. The projected surface areas of anode and cathode were 7 cm^2^ and the total MFC volume was 12 mL. The MFCs were electrically connected in one of two ways to examine the biofilm growth and conductivity (Fig 1). Double anode MFCs labeled ‘DA-MFC’ contained anodes in which both halves of the split anode were connected to the closed electrical circuit, while in single anode MFCs labeled ‘SA-MFC’ only one of the two halves of the split anode was connected to the closed electrical circuit. Reactors with neither of the two halves of the split anode connected to circuit were used as a control.

Lab-maintained active MFC cultures were used as inoculum, which was originally from active sludge collected from the Corvallis Wastewater Treatment Plant (Corvallis, OR) and has been demonstrated to converge to a consistent community composition over extended periods of operation and across varying reactor designs when fed acetate [27]. 30 mM of acetate was provided as the electron donor during the startup of MFCs which was increased to 60 mM after power outputs became stable. Modified *Geobacter* medium (MGM) (pH 7) was used in all experiments. The medium consists of the following ingredients (per liter): KCl, 0.13 g; NH_4_Cl, 0.31 g; NaH_2_PO_4_·H_2_O, 5.84 g; Na_2_HPO_4_·7H_2_O, 15.5g; vitamin, 12.5 mL; and mineral 12.5 mL solution as previously reported [22]. MFCs were operated in fed-batch mode with external resistance decreased from 10,000 to 500 Ω between batches as the biofilms grew in order to maintain maximum cell voltages around 0.3 V. When voltages were under 5% of batch maximum, the media was removed and replaced with new media. The length of each batch was approximately 48 hours. Though activated carbon air-cathodes were used, anodic growth was considered to be anaerobic in nature [28].

### Conductivity measurements

Electrochemical analyses were performed to measure the in situ resistance/conductivity of biofilms. For all experiments a conventional three electrode configuration was used in which a split anode was used as the working electrode, a 5% platinum plate (6.45 cm^2^) parallel to the split anode was used as the counter electrode, and a Ag/AgCl (3M NaCl) electrode was used as a reference. One half of the split anode was used as the electron source and the other half of the split anode was used as electron drain.

Initial experiments were conducted in order to evaluate biofilms over time at open circuit anode potential (OCP). In order to perform OCP conductivity measurements and to prevent interference from anodic current, the MFC anodes were temporarily disconnected from the cathode and allowed OCP to be reached. Then a voltage ramp (V_app_) low enough to avoid the electrolysis of water or a self-heating effect was then applied between two halves of a split anode (0–0.075 V) in steps of 0.025 V by using a source meter (Model 2405, Keithley, USA). The potential of both halves of the split anode were then continuously measured through the use of reference electrodes (Ag/AgCl, 3M NaCl). The average of the potentials of the two anode halves was considered the midpoint potential (E_mid_) of the biofilm, which was determined to be stable at -460 to -470 mV (vs. Ag/AgCl) in the presence of acetate containing media during open circuit conditions and when voltage ramps were applied (S1A Fig). For each voltage step, transient ionic current related to counter-ion diffusion was allowed to decline for approximately 20 minutes until a steady state was reached [8,29]. Current was then recorded every 30 seconds over a period of 3 minutes. Biofilm resistance was calculated by plotting V_app_ against measured current thereby avoiding the measurement of catalytic current associated with acetate oxidation. Conductance was then calculated from the inverse of resistance. Measurements were taken approximately twice a week during operation. Measurements of conductivity conducted at OCP were performed in triplicate reactors (n = 3).

To further measure biofilm resistance as a function of potential and to further examine conductive behavior of mixed species biofilms in the presence and absence of electron donor, electrochemical gating analysis was performed based on the three electrode configuration [15,30]. A potentiostat (References 100, Gamry Instruments Inc., Warminster, PA) in conjunction with the three electrode setup previously described was used to set the gate potential (V_g_), which ranged from -600 to 200 mV (vs. Ag/AgCl) [15]. Vg was increased in increments of 100 mV from -600 to -500 mV, 25 mV from -500 to -400 mV, 50 mV from -400 to -200 mV, and 100 mV from -200 to 200 mV. Concurrent with the setting of Vg, a source meter (Model 2405, Keithley, USA) was used to apply voltages (V_app_) between the source and drain anode. The conducting currents at various V_app_ (0, 25, 50, and 75 mV) were measured and used to calculate resistance from the slope of the voltage-current curve as previously described in preceding paragraphs. Following changes to V_g_ or V_app_, 20 minutes were waited before measurements were taken to allow the dissipation of transient ionic current. The changes of E_mid_ can be seen in S1B Fig. To measure biofilm conductivity in the absence of substrate, acetate-containing growth media was replaced with MGM containing no acetate to investigate conductive behavior in the absence of electron donor. Cell voltages of the MFCs dropped below 0.001 V within 24 hours following acetate removal and conductivity measurements were conducted by using two electrode method described above. Liquid samples were also collected and analyzed using high performance liquid chromatography (HPLC) to confirm that acetate was completely removed following medium replacement. Additional control experiments were also conducted. These controls included experiments with a biofilm-less anode using MGM with acetate, MGM without acetate, and sterile-filtered effluent from active MFCs. For all electrochemical gating experiments, deoxygenated media were prepared by bubbling of nitrogen gas for 30 minutes. Experiments of electrochemical gating analysis were conducted using multiple reactors (n = 2).

### Confocal laser scanning microscopy

Confocal laser scanning microscopy (CLSM) was used to verify biofilm growth across non-conductive gaps and on electrode surfaces in addition to measuring biofilm thickness. Following biofilm maturity, small pieces of the anodes (approximately 7 to 14% of the total anodic surface area) were carefully cut, stained with LIVE/DEAD BacLight Bacterial Viaibility Kit (Molecular Probes, Eugene, OR, USA), and then examined with the Zeiss LSM 510 Meta confocal microscope with a 10X objective lens (Carl Zeiss AG, Oberkochen, Germany). A minimum of three fields were imaged. Images were further processed and analyzed by software ImageJ (ver. 1.49d). A minimum of ten random CLSM stacks along the y-axis were used to determine the average thickness of biofilm.

### Fluorescent in situ hybridization

Fluorescent in situ hybridization (FISH) was used to confirm the presence of *Geobacter* spp. within the biofilm using the Geo1A probe [31]. For FISH analysis, a small piece of biofilm containing anodes was cut out as described above. Select pieces were fixed for 1–2 hours in 4% paraformaldehyde/ phosphate buffered saline (PBS) in a 24-well cell culture plate and subsequently permeablized by treatment with lysozyme (70,000 U/ml) in buffer for 10 min at 37°C then rinsed with PBS. FISH was performed immediately after fixation following procedures previously described [31]. Conditions were optimized with pure cultures of non-target strains and cultures of the target species by gradually increasing the formamide concentration in the hybridization buffer while maintaining equivalent ionic strengths and hybridization temperatures. Biofilms were first incubated in hybridization buffer consisting of 45% formamide, 20 mM Tris-HCl (pH 7.5), 0.9 M NaCl, and 0.01% SDS for 15 min at 46°C, and then immersed in the same solution containing the Geo1A probe (2 μg/ml) for 3 hrs at 46°C. Immediately following hybrization biofilms were soaked for 15 min at 48°C in the washing buffer (Tris-HCl (pH 7.5), 0.03 M NaCl, 0.01% SDS, 5 mM EDTA). Biofilms were rinsed in saline, lightly dried, and then embedded in a Citiflour antifadent (London, UK) before being stored in the dark at room temperature between 3–48 hours prior to examination. Samples were then examined by the Zeiss LSM 510 Meta confocal microscope with 10X and 20X objectives. Images were processed using ImageJ software (ver. 1.49d). A minimum of three fields were selected to represent the whole examined area.

### Biofilm conductivity calculation

Conformal mapping was used to calculate biofilm conductivity (*σ)* from biofilm thickness (*g)*, determined through CLSM measurements, and biofilm resistance (*R*), determined through two-electrode measurements [8,32]. The following equation was used, where *D* is the diameter of the electrode and *a* is the half of the width of the non-conductive gap:

σ=πRDln(8gπa)

### Cyclic voltammetry

Cyclic voltammetry (CV) was used to confirm direct electron transfer processes and identify major redox peaks in the mixed species biofilms. Following growth, a three-electrode electrochemical cell setup was used. The anode containing a biofilm was used as the working electrode and a platinum plate (6.45 cm^2^) was used as the counter electrode. An Ag/AgCl (3 M NaCl) was used as the reference electrode. For DA-MFCs, CV was performed in the presence and absence of acetate with substrate depletion confirmed through HPLC analysis. Biofilm-less control experiments were also conducted by filling control cells with the filtered MFC effluent. All CV experiments were performed by using a potentiostat (References 100, Gamry Instruments Inc., Warminster, PA) over a potential range from -600 to 200 mV vs. Ag/AgCl at a scan rate of 1 mV s^-1^. For all CVs, deoxygenated media was prepared by bubbling nitrogen gas for 30 minutes.

## Results

### Growth and spatial distribution of biofilms

**Fig 2:**
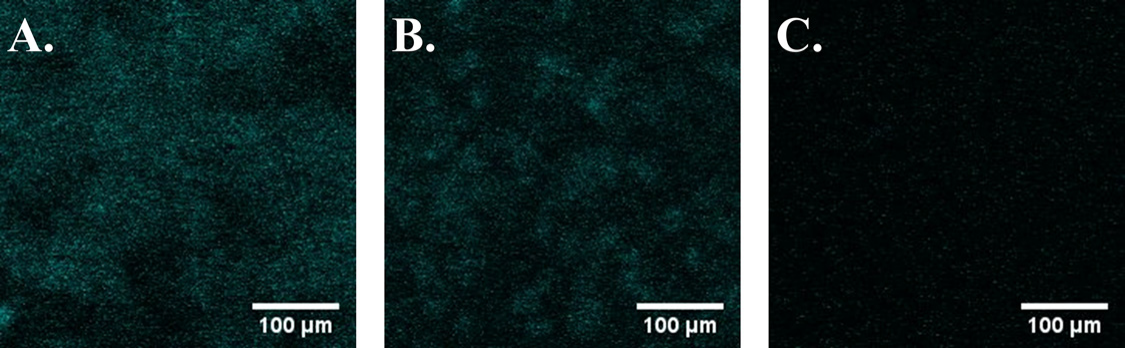
Fluorescent in situ hybridization images displaying *Geobacter* distribution. (A) On actively respired anode surface, (B) On non-actively respired surface of the same MFC, (C) On surface of control MFC. Frame size = 1.6 X 10^−3^ cm^2^.

CLSM imaging revealed that biofilms formed over the 50 μm non-conductive gaps between the two active anode surfaces connected to the circuit in DA-MFCs. Average biofilm thickness within the gap area of DA-MFCs was 38.4 ± 10.1 μm after approximately 70 days of growth (Fig 1A). Biofilm growth was also observed across the non-conductive gap in SA-MFCs with an average biofilm thickness of 52.2 ± 11.6 μm within the gap area (Fig 1B). Biofilm growth on the unconnected anode surface (~1 cm away from an active electrode surface) was observed in all anodes of SA-MFCs with less red pigment than active electrode surfaces (S2 Fig). Control reactors with unconnected anodes displayed limited growth on gold-coated electrode surfaces and biofilms were too sparse for thickness to be measured (Fig 1C). *Geobacter* spp. were detected on both active and inactive anode surfaces of DA-MFCs and SA-MFCs but not detected on any electrode surfaces of control reactors (Fig 2).

### Current and power generation

**Fig 3:**
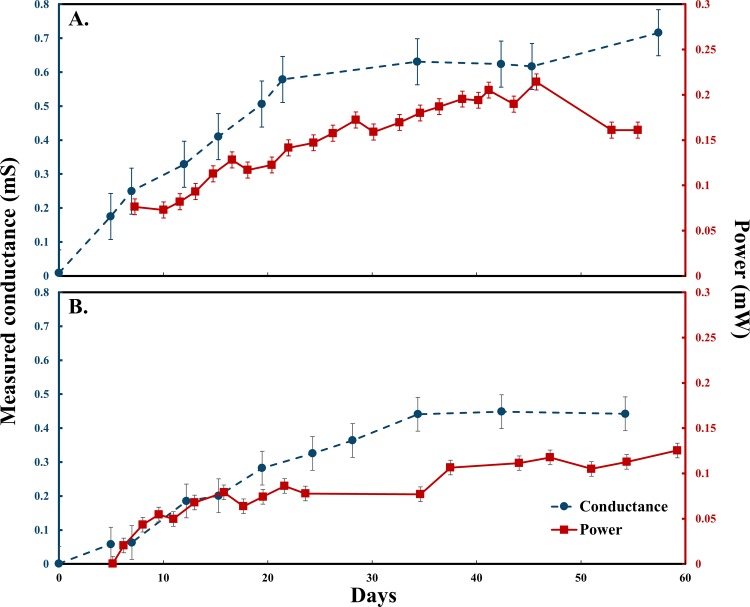
Measured conductance and power of DA-MFCs and SA-MFCs over time. (A) DA-MFC with 50 μm non-conductive gap, (B) SA-MFC with 50 μm non-conductive gap. Error bars represent standard deviation (n = 3).

Increases over time in electrical current and power generation were observed for both DA-MFCs and SA-MFCs (Fig 3). When normalized to cathode surface area, power generation of SA-MFC reactors averaged 63.1% of the power generation of DA-MFC reactors, significantly greater than the 50% that would be expected if no exoelectrogenic growth was on the unconnected electrode surface (single factor t-test, α = 0.05). This observation suggests that the establishment of an electrical connection beyond the active electrode was simultaneously supported by the growth of exoelectrogens on the inactive anode surface of SA-MFCs and contributed to power generation.

### Conductivity of mixed-species biofilms

Initial conductance was calculated during growth at biofilm mid-point potentials (E_mid_) of -470 mV (vs. Ag/AgCl) corresponding to open circuit potentials of anode. Conductance was observed in both DA- and SA-MFC reactors and increased as biofilms grew across non-conductive gaps (Fig 3). Initial increases in power were correlated to increases in conductance yet continued to increase after conductance plateaued. This is likely due to stacking of active biomass enabled by the conductivity of the inner biofilm structure. Average biofilm conductivity for DA-MFCs was 679.4 ± 15.6 μs cm^-1^. This conductivity is significantly higher than that calculated for the open-circuit control reactor using average biofilm thickness of DA-MFCs (3.2 ± 1.5 μs cm^-1^). Increases in conductance proceeded slower in SA-MFC setups likely due to a single point of origin for exoelectrogenic growth across the gap (Fig 3B). The average conductivity of SA-MFC reactors was 285.2 ± 52.3 μs cm^-1^ at the same E_mid_, corresponding to a 58.2% decrease compared to DA-MFCs.

**Fig 4:**
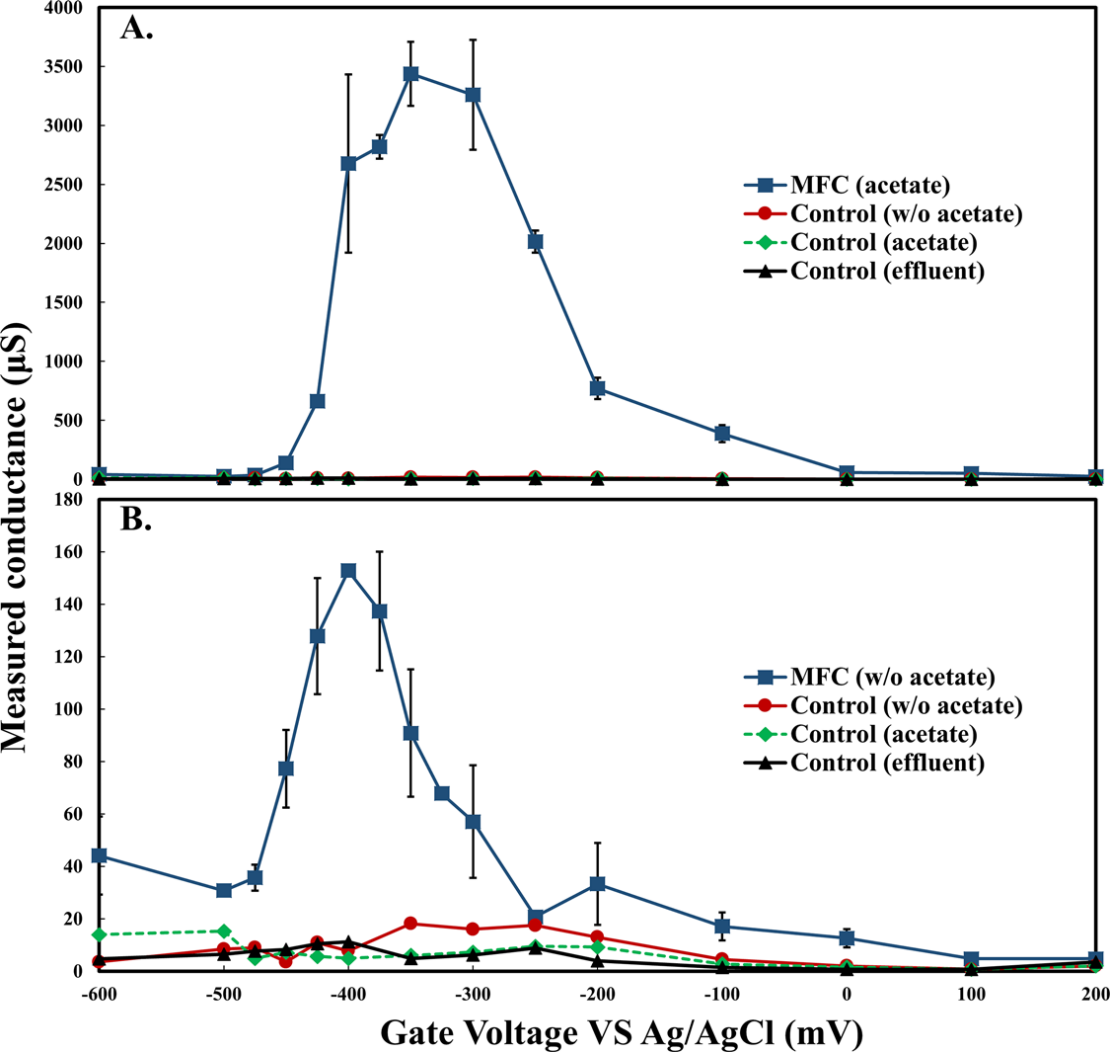
Conductivity of mixed species biofilms and control estimated by electrochemical gating analysis. Error bars represent standard deviation (n = 2).

After the conductivity plateaued, conductivity was then further analyzed over a range of potentials using electrochemical gating analysis. Results revealed that the conductivity of mixed species biofilms changed in a peak-manner based on gate potential (V_g_) (Fig 4A). Maximum conductance of the mixed species biofilms was 3437 ± 271 μS corresponding to a V_g_ of -350 mV (vs. Ag/AgCl). Conductance of biofilms poised at a V_g_ more negative than -500 mV or more positive than 0 mV were significantly decreased. Measurements in both control experiments using the same electrode geometry but without established biofilms did not exhibit a peak in conductivity and only marginal conductivity was observed (0.8 to 15.3 μS), indicating that the observed conductivity was related to the development of an exoelectrogenic biofilm.

To investigate the effect of electron donors on biofilm conductivity, measurements were also performed in the absence of acetate. Following the removal of acetate from the growth media, electrical current production ceased (S3 Fig). Peak conductance estimated through electrochemical gating analysis shifted from -350 mV to -400 mV (vs. Ag/AgCl) while maximum conductance decreased from 3437 ± 271 μS to 153 ± 2 μS (Fig 4B). After acetate was added back to the growth media current production in each reactor recovered immediately indicating biofilms remained intact during acetate removal testing (S3 Fig).

### Cyclic voltammetry

**Fig 5:**
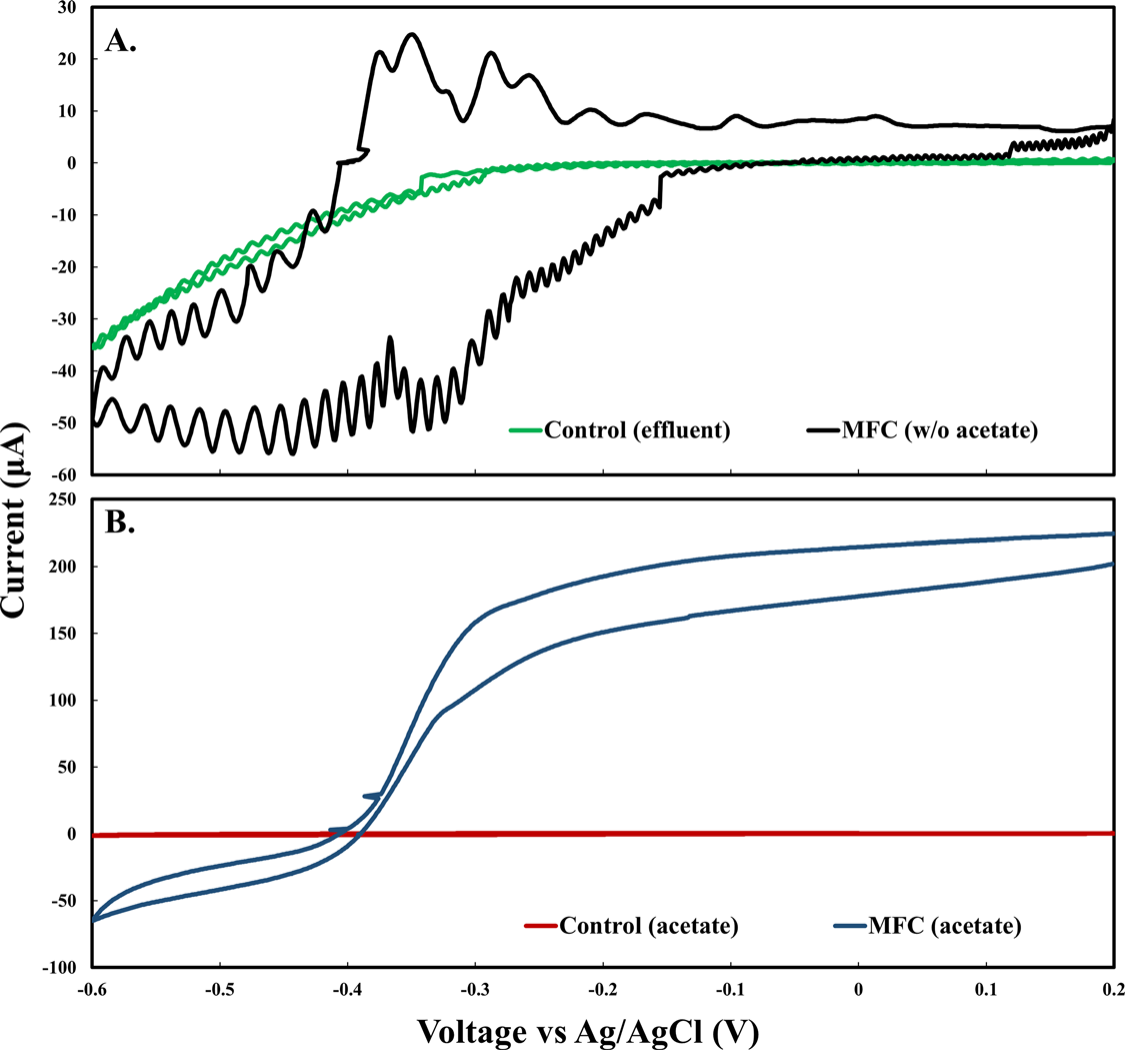
Cyclic voltammograms of mixed species biofilm. The voltammograms were recorded (A) in the absence of electron donor and (B) in the presence of electron donor. Scan rate was 1 mV S^-1^.

In the absence of electron donor, two major redox peaks were identified at potentials of -370 and -310 mV (vs. Ag/AgCl) (Fig 5A). Multiple additional peaks from -300 to -600 mV (vs. Ag/AgCl) were also detected suggesting the existence of multiple redox cofactors spanning a range around formal potentials similar to what have been observed previously [18,33]. No redox peaks were observed in the control electrochemical cell (biofilm-less anode) with filtered MFC effluent. These results indicate that observed current can be attributed to the altering of the oxidation state of these redox cofactors localized in present biofilms and electrochemical activity can be attributed to membrane-bound redox cofactors like outer membrane cytochromes (OMCs) as opposed to extracellular electron shuttles [18]. In the presence of acetate, the midpoint of catalytic current associated with the oxidation of acetate occurred at -360 mV (vs. Ag/AgCl) similar to the primary peaks observed in the absence of acetate (Fig 5B). In biofilm-less reactors no current response was observed.

## Discussion

### Properties of mixed-species biofilms

The most significant difference between previous studies featuring pure cultures and the present one featuring a mixed species community is the response of conductivity to the removal of an electron donor. In previous studies using single species exoelectrogenic biofilms were not significantly influenced by the removal of acetate [8]. However, in the present study, when acetate was removed from the medium decreases in peak conductivity as large as 96.6% were observed along with simultaneous -50 mV shift in biofilm potential at which maximum conductivity occurs. These differences reflect changes in the type and activity of localized redox cofactors as well as magnitude of electron accumulation within the biofilm [17,34]. Another difference between previous studies featuring single species biofilms and the mixed species biofilms of the present study is the ability to establish growth on an inactive electrode 1 cm away from an active electrode (Figs 1 and 2) [8,24]. The extended growth in the mixed-species environment was possible due to the the presence of alternate redox factors and the likely presence of interspecies coadhesion interactions that enable extended growth distances from active electrodes [6,35–37].

### Conductive mechanisms

Further investigation into the conduction mechanism of mixed species exoelectrogenic biofilm was conducted by measuring biofilm conductivity as a function of potential. A conductivity peak was observed around a V_g_ of -360 mV (vs. Ag/AgCl), similar to the formal potential of c-type cytochromes associated with other exoelectrogenic biofilms [38]. The peak-manner response observed is a characteristic of a redox molecule-mediated electron transfer mechanism like that of conductive polymers [29,39]. Classically, this electron transport is considered to be driven by concentration gradients of localized reduced and oxidized redox cofactors though voltage gradients may also be responsible. Either way the fundamental electron hopping event is unchanged and can be unified under generalized thermodynamic forces [29]. Concentration gradient driven electron transfer is dependent on a mixed valent state and maximized at potentials in which the ratio of reduced and oxidized redox sites within the matrix are equal [29]. If redox sites are completely reduced or oxidized within the biofilm matrix, the biofilm will become non-conductive as observed in the present study when V_g_ was more positive than 0 mV or more negative than -500 mV. Overall support of a concentration gradient driven conduction mechanism in the present study is consistent with previous research presenting an electron hopping model of electron transfer in single species biofilms of *G*. *sulfurreducens* [17–19].

### Biofilm conductivity evaluation methods

Despite the importance of constructing conductive biofilms/aggregates as direct extracellular electron transfer conduits, few studies have been published related to conductive biofilms/aggregates and their conduction mechanisms. In the present study, a practical two-electrode setup was used to allow the measurement of conductive current in microbial biofilms. Experiments were designed to ensure accurate measurements of conductivity of biofilms. First, the large conductive surface areas of the two electrodes (3.5 cm^2^) combined with a thin non-conductive gap (50 um) ensured proper biofilm development on the active electrode surfaces and the nonconductive gaps, allowing the measurement of conducting current. Second, steady state experiments were used to avoid measuring transient ionic current [29]. In the case of redox gradient driven electron conduction, the self-exchange of electrons between the redox sites is accompanied with the diffusion of counter-ions for the sake of electroneutrality [29]. Third, appropriate means were employed to account for catalytic current related to acetate oxidation [17]. In addition to removing the electron donor during electrochemical gating analysis, the slope of V_app_ versus current was also used in the calculation to avoid the inclusion of the catalytic current.

Additional tests to further elucidate conduction mechanisms of biological materials include examining redox potential dependency, temperature dependency, pH dependency, and behavior following exposure to several inhibitors [8,22]. However, many of these experiments might induce destruction of the delicate biofilm structure and should be carefully conducted to ensure the intactness of biofilms. Electrochemical gating analysis used in present study is relatively moderate redox gradient dependency test, and should be carried out within biological range based on previous observations [24,30]. The relationship between biofilm conductivity and electrochemical potential also suggests that biofilm conductivity should be reported along with the potential at which the measurement was performed.

## Conclusions

Mixed-species communities will continue to play a critical role in many MESs. The current study provides an initial reference point for the conductive behavior of mixed-species biofilms. Differential responses were observed in the absence of an electron donor and over varying potentials suggesting redox driven conductivity of the mixed-species exoelectrogenic biofilms. The construction of these conductive biofilms is clearly one strategy that communities use to effectively take advantage of inexhaustible, insoluble electron acceptors in addition to also providing a conduit for direct electron transfer that is the foundation of many synergistic relationships. Further understanding the conductive behavior and electronic interactions of these communities will lead to improvements of power and efficiencies in MESs, and may potentially open up new bioelectronics applications.

## Supporting Information

S1 Fig Changes of midpoint potentials during experiments. (A) two-electrode experiment (B) electrochemical gating analysis.
(TIF)

S2 Fig Top down digital camera pictures. (A) Anode from DA-MFC, (B) Anode from SA-MFCs, and (C) Bare surface of gold electrode.
(TIF)

S3 Fig Acetate removal experiment. Blue round dots represent the current of MFC and red solid line represents the acetate concentration detected by HPLC.
(TIF)
