# Supplementary figures and images for: Redox Conductivity of Current-Producing Mixed Species Biofilms
Source: PLoS One. 2016 May 9;11(5):e0155247. doi: 10.1371/journal.pone.0155247 (PMC4861276; doi:10.1371/journal.pone.0155247)

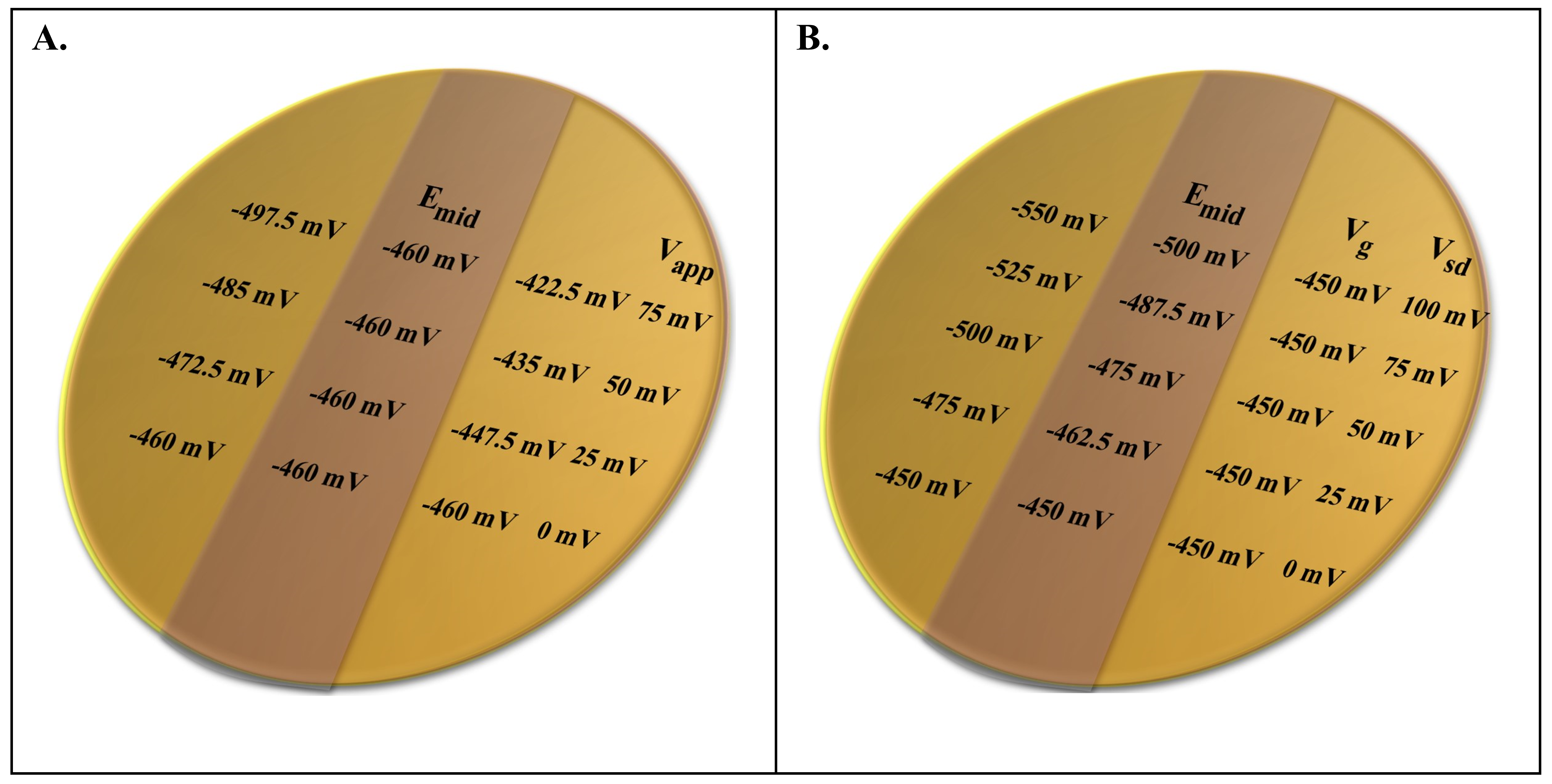

Supplement: S1 Fig — (A) two-electrode experiment (B) electrochemical gating analysis. (TIF) [file pone.0155247.s001.TIF]

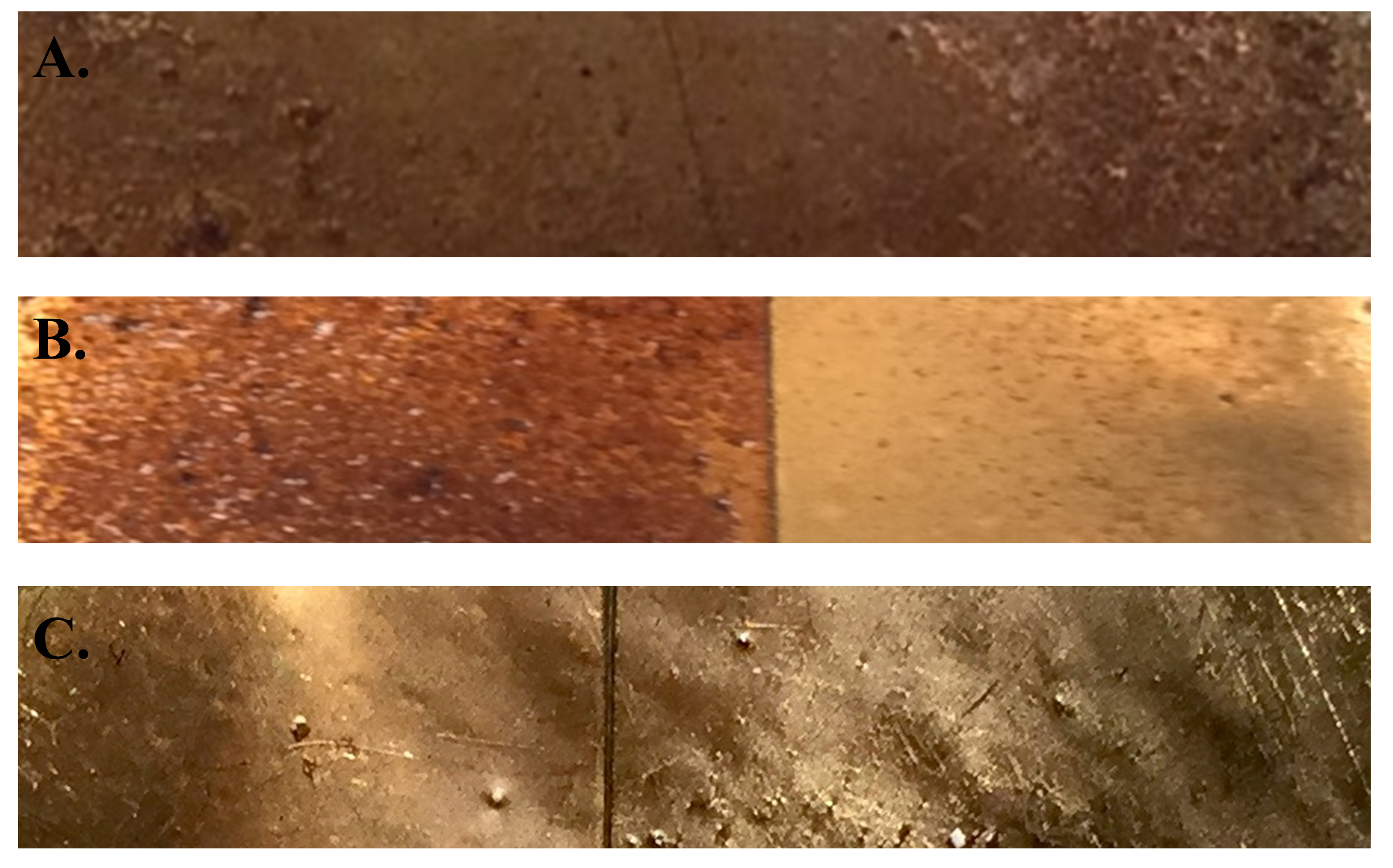

Supplement: S2 Fig — (A) Anode from DA-MFC, (B) Anode from SA-MFCs, and (C) Bare surface of gold electrode. (TIF) [file pone.0155247.s002.TIF]

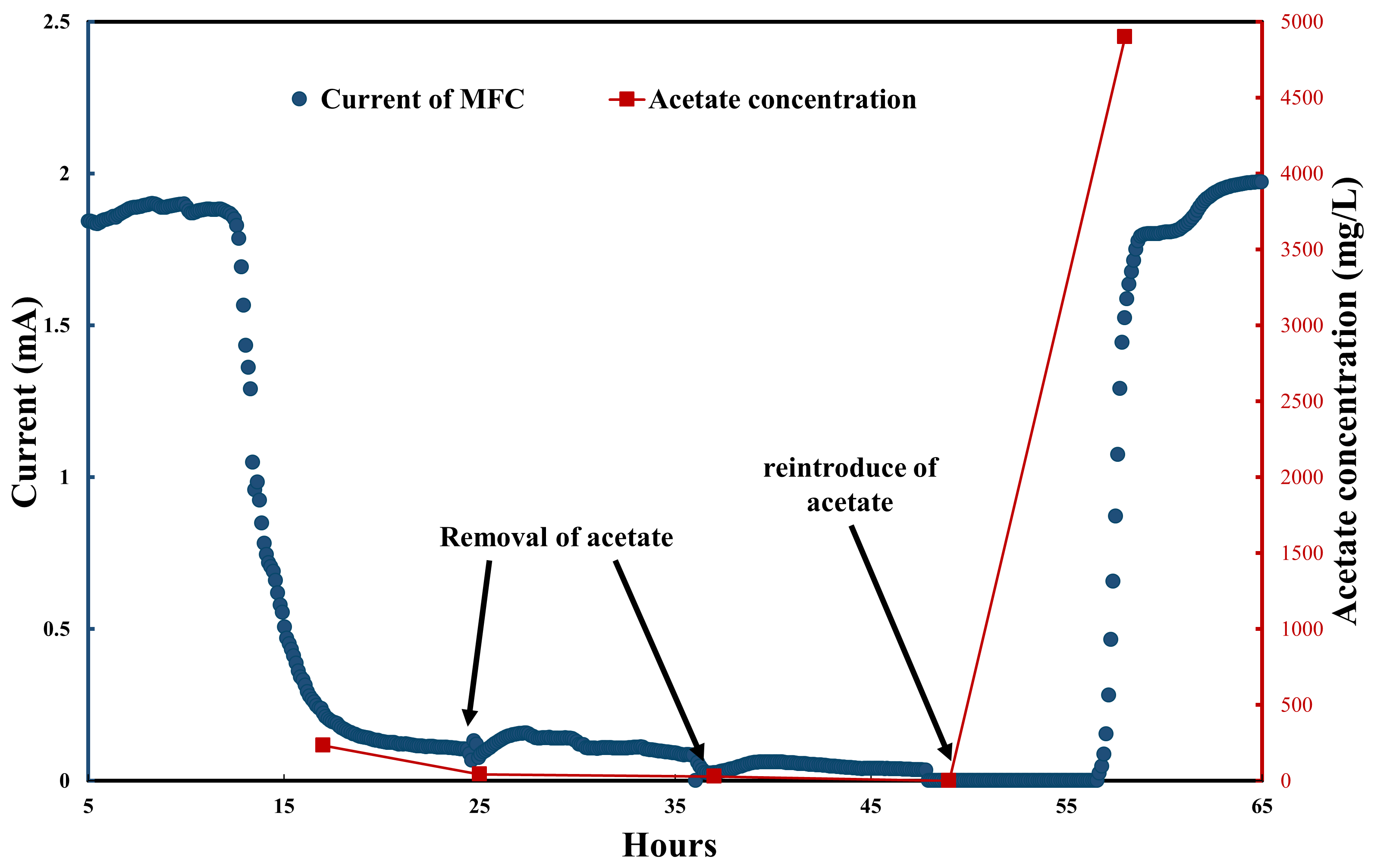

Supplement: S3 Fig — Blue round dots represent the current of MFC and red solid line represents the acetate concentration detected by HPLC. (TIF) [file pone.0155247.s003.TIF]
